# Practice and Learning: Spatiotemporal Differences in Thalamo-Cortical-Cerebellar Networks Engagement across Learning Phases in Schizophrenia

**DOI:** 10.3389/fpsyt.2016.00212

**Published:** 2017-01-23

**Authors:** Michele Korostil, Gary Remington, Anthony Randal McIntosh

**Affiliations:** ^1^Centre for Addiction and Mental Health, Toronto, ON, Canada; ^2^Rotman Research Institute of Baycrest Health Sciences, Toronto, ON, Canada; ^3^University of Toronto, Toronto, ON, Canada

**Keywords:** schizophrenia, fMRI, learning, practice, cognition, multivariate, longitudinal, partial least squares

## Abstract

**Background:**

Understanding how practice mediates the transition of brain–behavior networks between early and later stages of learning is constrained by the common approach to analysis of fMRI data. Prior imaging studies have mostly relied on a single scan, and parametric, task-related analyses. Our experiment incorporates a multisession fMRI lexicon-learning experiment with multivariate, whole-brain analysis to further knowledge of the distributed networks supporting practice-related learning in schizophrenia (SZ).

**Methods:**

Participants with SZ were compared with healthy control (HC) participants as they learned a novel lexicon during two fMRI scans over a several day period. All participants were trained to equal task proficiency prior to scanning. Behavioral-Partial Least Squares, a multivariate analytic approach, was used to analyze the imaging data. Permutation testing was used to determine statistical significance and bootstrap resampling to determine the reliability of the findings.

**Results:**

With practice, HC participants transitioned to a brain–accuracy network incorporating dorsostriatal regions in late-learning stages. The SZ participants did not transition to this pattern despite comparable behavioral results. Instead, successful learners with SZ were differentiated primarily on the basis of greater engagement of perceptual and perceptual-integration brain regions.

**Conclusion:**

There is a different spatiotemporal unfolding of brain–learning relationships in SZ. In SZ, given the same amount of practice, the movement from networks suggestive of effortful learning toward subcortically driven procedural one differs from HC participants. Learning performance in SZ is driven by varying levels of engagement in perceptual regions, which suggests perception itself is impaired and may impact downstream, “higher level” cognition.

## Introduction

Learning impairments in schizophrenia (SZ) are widespread, impact functional outcome, and may mediate response to various types of cognitive rehabilitation strategies. Despite considerable efforts to remediate cognitive impairments *via* cognitive training, pharmacologic and brain stimulation treatments, effect sizes thus far have been modest (1, 2). The unknown aspects of the neural dynamics of learning and the impact of practice in SZ continue to constrain the search for the optimal therapeutic brain targets. Prior practice-related neuroimaging experiments in SZ have relied primarily on single scanning sessions and univariate, task-related (vs. behavior-related) analyses (3–8). However, brain substrates associated with practice-related learning are dynamic and widely distributed in space and time. Thus, to measure these substrates using fMRI, one should use multivariate whole-brain imaging analytic techniques, a direct brain–behavior (vs. brain–task) approach to analysis, and multiple scanning sessions with sufficient in-scanner practice to measure the unfolding of brain changes *in vivo*.

In healthy control (HC) individuals, practice-related learning typically follows a characteristic staged process: early, rapid, and effortful learning engaging widespread cortical–subcortical areas with a later shift to more automatic processes and a reduced number of prefrontal regions (4, 5, 9–13). This process is characteristic of both motor skill and cognitive skill-based learning where ongoing practice leads to incremental learning of associations. Similarly, dual-processing models of practice-related learning postulate a domain-general cognitive-control network that scaffolds early learning, but gradually disengages with practice as skilled, automatic processing emerges (14, 15). As per this model, this shift occurs in various types of experience-driven learning as long as learning is intentional with the specific details varying depending on task requirements (14). In SZ, this shift is less clear. Learning remains more effortful throughout with persistent cortical involvement at stages when HC persons have transitioned to subcortically driven automatic stages of processing. This lack of transition occurs both in SZ (on and off antipsychotics) and in those at genetic high risk for the disorder (16, 17). Persistent effortful learning also may reflect impairments in deep encoding in the disorder, particularly in verbal memory and relational-learning paradigms (18, 19).

Several studies examining practice-related learning in SZ have noted exponential blood oxygenation level-dependent (BOLD) signal decrease in task-relevant brain areas, which has been primarily driven by initial hyperactivity in these same regions (3–7). Koch et al. found no neural differences in task activation between successful learners with SZ and HC during a Sternberg working memory task. However, using a median split, the low-performing participants with SZ were more likely to show initial hyperactivation in frontal, cingulate, and superior-parietal regions (7). Activation studies using associative learning tasks have found both increased and similar levels of brain activation in SZ (20, 21). Proposed explanations for aberrant activation patterns in learning studies have included neural inefficiency and alternative compensatory pathways to achieve behavioral parity (8, 22). We have also suggested that some of these results could be attributable to non-specific task-independent effects given the potential linear confound of time inherent in learning studies in SZ (23).

Thus, while many studies have examined putative compensatory brain strategies for learning impairments in general and some have examine the impacts of practice, none, to our knowledge, have utilized an in-scanner multi-day practice paradigm that allows for direct observation of the unfolding of learning as it occurs *in vivo* across a number of learning phases. Our experiment was a multisession fMRI scanning experiment wherein participants with SZ and HC participants learned a novel lexicon with in-scanner practice over the course of 1 week. We utilized a lexicon-learning relational paradigm because verbal learning impairments are common in the disorder and are particularly linked to functional outcome at all stages of the illness (24–27). Additionally, language disorders are hallmark phenomenological features at all stages of the syndrome (28). We used behavioral-PLS analytic techniques, which allowed for direct measurement of the brain–behavior relationship. This approach allowed us to examine the large-scale distribution of learning in the brain as it unfolded over the course of practice and avoided the previously mentioned confounding effects of task-independent BOLD changes, common contaminants in practice-related learning neuroimaging experiments that we examined specifically in SZ in an earlier analysis of this study data (23).

## Materials and Methods

### Participants

Participants were 16 patients with DSM-IV diagnosed SZ recruited from the outpatient clinics at the Centre for Addiction and Mental Health (CAMH) matched with 17 HC recruited *via* local advertisement and a research participant database at Baycrest Hospital. Both are teaching hospitals associated with the University of Toronto. All participants were right handed (29), native English speakers, and suitable for MRI scanning. Participants were comprehensively screened for and excluded if there were any interfering medical conditions, neurological disorder, or psychiatric disorder. Participants with SZ were clinically stable and had been prescribed an atypical antipsychotic medication at a stable dose for at least 3 months. The diagnosis of SZ was confirmed and other Axis 1 psychiatric disorders ruled out by the study MD (Michele Korostil) using the Mini-International Neuropsychiatric Interview-Plus (30). The clinical status of the SZ participants was assessed using the Positive and Negative Syndrome Scale (PANSS) (31) at the initial visit and the Clinical Global Impressions Scale (32) at all three study visits. The neurocognitive status of all participants was evaluated at the initial visit using the Repeatable Battery for the Assessment of Neuropsychological Status (RBANS) (33). Five HC and four SZ participants were excluded from the final analysis: two due to improper task performance, three due to technical difficulties with equipment, and four due to excessive movement artifact on MRI scans. Thus, the final sample included 12 matched participants from each group.

The study protocol was approved by the Research Ethics Boards of Baycrest Hospital and the CAMH according to guidelines from these hospitals and the University of Toronto. Participants provided written informed consent and were paid a stipend for their participation.

### Study Procedure

The study occurred over a 3-day period. On day 1, participants were assessed for suitability, and the PANSS and RBANS were administered. Participants were trained on the fMRI learning task in an MRI simulator using a parallel set of stimuli. On days 2 and 3, participants completed the fMRI experiment in which they learned a 30-word novel lexicon while undergoing fMRI scanning. The structure of the sessions on days 2 and 3 were identical. The vocabulary was the same for both days and thus day 3 functioned as a “practice session” to consolidate the learning from the prior session. The mean interval between scanning sessions for all participants was 2 days.

### Stimuli Selection and Task Design

**Figure 1:**
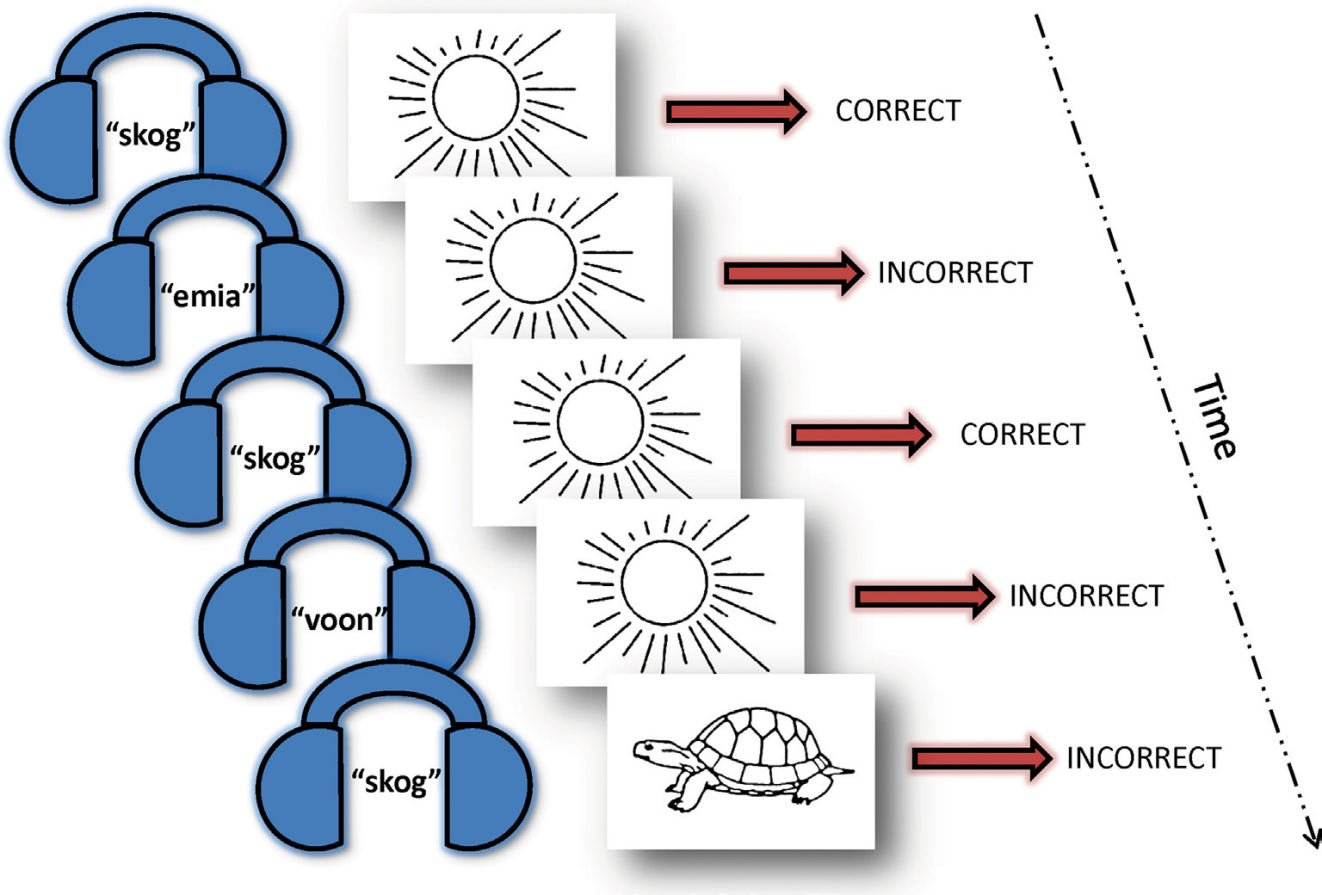
**Vocabulary learning task**. Over the course of 10 “learning runs” (five runs on day 1 and five runs on day 2) with 1,200 trials total, each pseudoword (here: skog) appeared 20 times paired with the same picture (here: sun) and only once with other varying pictures. Participants were instructed that they would be learning a new vocabulary, but were not informed of the underlying learning principle and did not receive feedback throughout the experiment. Thus, they intuitively learned that the recurring couplings were the correct pairs. A 30-word picture-word vocabulary was randomly generated for each participant using a normed set of English pseudowords paired with a standardized picture of an everyday object. Each participant’s vocabulary remained the same for both scanning sessions. They indicated their response (“correct” or “incorrect” pairing) *via* a button press while in the fMRI scanner.

Participants were asked to learn a novel 30-word vocabulary comprised of auditory English pseudowords arbitrarily paired with pictures of everyday objects while undergoing event-related fMRI scanning. The task was developed by Breitenstein and Knecht (34, 35) for German speakers and modified by us for native English speakers. In our version of the task, participants were informed in advance that they would be learning a new vocabulary over the course of the experiment. All participants were trained on the task in an fMRI simulator using a parallel set of stimuli to 75% proficiency to minimize contamination by “learning the task” during fMRI data acquisition. While lying prone in the MRI scanner, participants heard a spoken pseudoword in their headphones (normalized to 600 ms duration). Shortly after (200 ms) the onset of the auditory pseudoword, they saw a picture of an object (duration 1 s) followed by a fixation cross (2 s). This sequence constituted one trial. Participants were asked to indicate, *via* a button press on a response pad, if the words and pictures went together in the new vocabulary. The pairings were sometimes “correct” and sometimes “incorrect.” While participants were informed at the outset that they would be learning a new vocabulary, there was otherwise no feedback given to participants throughout the experiment. The “correct” pairings repeated throughout the experiment, each “incorrect” pairing was presented only once. Thus, the underlying learning principle was a higher cooccurrence of “correct” trials with a 20:1 (correct:incorrect) ratio by the end of both scanning sessions (Figure 1). The lexicon was randomly generated from a matched set of stimuli and different for each participant. Each participant’s lexicon remained the same across the scanning days and, thus, the learning runs were additive. Each day of learning in the scanner involved 5 “learning runs” (approximately 6 min in length), which were each comprised of 120 trials as described above for a total of 1,200 trials over the course of the entire experiment with half being “correct” pairings and the other half “incorrect.”

### fMRI Data Acquisition and Preprocessing

fMRI was performed on a research-dedicated Siemens Trio 3-T MRI scanner. T2* functional images (TE = 30 ms, TR = 2,000 ms, flip angle = 70°, FOV = 200 mm, in-plane voxel size = 3.5 mm × 3.5 mm × 5.0 mm) were acquired using a single shot echo-planar image sequence leading to a BOLD contrast. Each functional sequence consisted of 28 5-mm thick slices in the axial-oblique plane positioned to image the entire brain. A T1-weighted anatomical scan was obtained using a SPoiled Gradient Recalled sequence (TE = 2.6 ms, TR = 2,000 ms, FOV = 256 mm, slice thickness = 1 mm) for coregistration with the functional images and to ensure that there were no significant brain abnormalities in any participants.

Participants’ responses were made on an MR-compatible button response pad (Fiber-Optic Response Pad system).^1^ Visual stimuli were viewed in a head-coil mounted mirror directed toward a rear projection screen at the foot of the scanner. Auditory stimuli were presented using the Silent Scan auditory presentation system (AVOTEC). E-Prime Software^2^ was used to control stimulus presentation and to collect behavioral responses.

The functional data were processed prior to statistical analysis. Slice-timing correction was done using AFNI.^3^ Motion correction was completed using AIR.^4^ The motion-corrected functional volumes within each run were averaged to create a mean functional volume per run. This mean functional run was then registered with each participant’s structural volume using a rigid body transformation model. The structural data were spatially normalized to the Common Anatomical Template and the end result was a direct non-linear transform from each initial fMRI volume into the Common Template space (36–38). Given the possible morphologic differences between the two groups, our approach helps protect against distortion that could be caused by the use of a standard space template based only on healthy brains. The functional data were then smoothed using a 7-mm Gaussian kernel. The voxel time series were further adjusted by regressing out motion correction parameters, white matter time series, and CSF time series, as in Garrett et al. (37).

Results from data analyses were transformed into MNI space using the FSL/FNIRT registration algorithm to find a non-linear transform between our anatomical template and the MNI152_T1 template provided with FSL software.^5^ We used SPM5^6^ to further assist with anatomical localization.

### Data Analysis

#### Behavioral Measures

Behavioral performance (accuracy and reaction time) during the fMRI experiments was analyzed using repeated measures ANOVAs.

#### fMRI Analyses

To identify multivariate patterns of relationships between performance and brain activity, we used Behavioral-Partial Least Squares (BPLS) (39). PLS is a multivariate statistical method that identifies maximal covariance [latent variables (LVs)] between sets of independent measures and allows for both spatial and temporal interpretation. It is mathematically similar to canonical correlation analysis. PLS analysis of neuroimaging data has been demonstrated as an effective method in SZ experiments with small samples and multicollinear measures (40, 41). BPLS is a type of PLS that allows for direct identification of multivariate patterns characterizing brain–behavior (in our case brain–accuracy) relationships rather than relying on inference *via* task performance. In BPLS, PLS solutions are constrained to the *part* of the covariance structure directly related to behavior. Importantly, the approach allows for analysis of all the relationships between performance on the task across two days of learning for all participants and BOLD signal in all gray matter regions in one model, without the need to restrict analysis to specific regions of interest.

A correlation matrix between accuracy for each learning run and the BOLD signal at each voxel for each correct trial across both fMRI scans (10 learning runs) was created for each subject. Given that the hemodynamic response function for each condition lasts for several scans, PLS utilizes a “lag window” (i.e., a short signal segment) to best capture the response of each voxel. The lag-window size was 8 (TR = 2, 16 s), beginning at the offset of the auditory pseudoword (600 ms from beginning of each trial in the learning runs). The event markers were set at the beginning of this lag window to best capture language-related activation (35). The correlation matrix was then decomposed using singular value decomposition to produce LVs that capture the optimal relationship between learning accuracy and BOLD signal across the entire brain. Essentially, behavior-PLS is mathematically identical to a multivariate seed-connectivity analysis wherein accuracy is the “seed” and the results demonstrate how accuracy covaries with brain activity across subjects during the experimental learning runs. Each LV contains the spatial pattern displaying brain regions where BOLD activity is mostly strongly related to learning performance for each group. The LVs consist of the correlation strength (i.e., the “singular value”) and the weighting pattern across all brain voxels that optimally expresses the correlation (i.e., the “brain saliences”). Brain scores, akin to component scores in PCA, are summary measures for each participant that shows the degree to which a participant expresses the multivariate spatial pattern captured by a given latent variable.

Significance testing of the resultant multivariate relationship between brain functional patterns and learning accuracy was assessed with 1,000 permutation tests of the singular value (SV) associated with each latent variable. As this operation is done on the entire data structure simultaneously, there is no need to correct for multiple comparisons at the voxel level. Bootstrap resampling (1,000 resamples) was used as a measure of the robustness of the voxel contribution to the effect.

Each voxel’s bootstrapped mean salience was divided by its estimated SE to obtain a normalized estimate of robustness [i.e., “bootstrap ratio” (BSR)]. A BSR of 3.00 approximates a 99% confidence interval. Our tables and images were thresholded at a BSR of 3.

Correlation matrices for the brain scores for the significant LVs across all 10 learning runs were constructed. High correlations that tracked across a series of learning runs would indicate consistent movement through the learning task for a given individual subject. For example, assuming consistent effort and a clear underlying learning principle of the task, behavioral performance for any given subject on any given run should correlate with performance on subsequent runs. Similarly, one could make the same assumptions for brain activity and deviations from this could indicate a change in brain “strategy” due to changes in the underlying learning processes.

The mean of the brainscores for a given series of runs that represented a coherent brain–behavior relationship was calculated for each individual for each significant LV. The mean was then correlated with symptom (PANSS), neuropsychological scores (RBANS), and antipsychotic medication dose (CPZ equivalents) (42) to better understand characteristics of participants with a given brain–behavior pattern. The approach also had the benefit of reducing the data so as to reduce risks of spurious conclusions inherent with multiple correlational analyses.

## Results

### Demographic and Clinical Characteristics

Table 1 displays demographic and clinical characteristics of the study participants. Participants were matched with regard to sex and age and were found to differ in years of education and additional languages spoken. The neurocognitive assessment showed significant differences between the groups on all subscales except for delayed memory. The SZ group was in the “mildly symptomatic” range of psychopathological symptoms as estimated *via* the PANSS (positive subscale = 11.8 ± 2.6, negative subscale = 9.5 ± 3.1, general subscale = 24.0 ± 3.6, and total = 45.2 ± 6.2) (43). Using Woods’ guidelines, the mean antipsychotic dose in chlorpromazine equivalents for the SZ group was 474 mg (42).

**Table 1:** **Demographics and neurocognitive tests**.

	HC	SZ	*p*-Value
Age (±SD)	30.8 ± 8.2	32.3 ± 10.6	0.54
Male/female (χ^2^)	7/5	8/4	0.67
Education (years)	17.3 ± 2.5	14.3 ± 4.1	0.039
Number languages spoken	2.1 ± 1	1.2 ± 0.4	0.007
WRAT	53 ± 3.3	50.2 ± 5.5	0.14
RBANS total (percentile scores)	67.6 ± 18.5	32 ± 28.5	0.002
Immediate memory	55.6 ± 29.8	29.8 ± 28.7	0.047
Delayed memory	50.9 ± 27.4	31.2 ± 20.4	0.062
Visuospatial/constructional	76.3 ± 20.9	49.2 ± 35.0	0.037
Language	57.6 ± 19.9	34.8 ± 26.3	0.03
Attention	68.6 ± 27.3	38.4 ± 34.4	0.03

*HC, healthy control group; SZ, schizophrenia group; WRAT, Wide Range Aptitude Test; RBANS, Repeated Battery for the Assessment of Neuropsychological Status*.

### Behavioral Data

**Figure 2:**
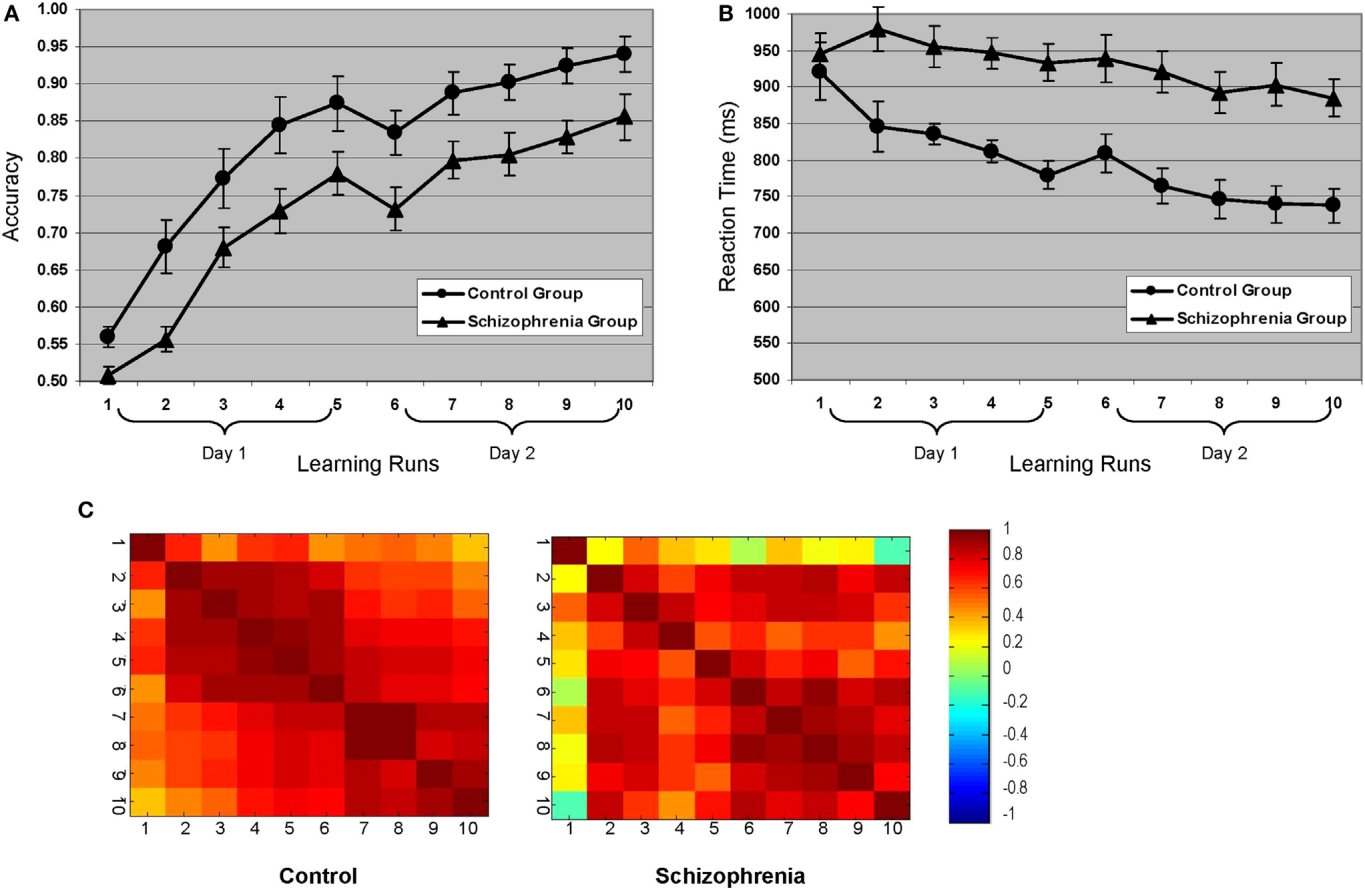
**Behavioral learning curves for both groups: (A) accuracy and (B) reaction time**. **(C)** Accuracy correlation matrices across all 10 learning runs for each group. The primary difference between the groups occurs in the first learning run, which is consistent with the finding that control participants began learning the lexicon in the first run, whereas the schizophrenia participants did not start acquiring the lexicon until the second run (see learning curves). Note that during the first learning run, there was 50% less “learning” opportunity than subsequent runs given that correct responses on the initial presentation of any given “correct” pairing would be due to chance.

There was a significant learning effect for both groups across the 10 learning runs [*F*(4, 89) = 116.43, *p* = 0.0001] (Figure 2). Both groups showed similar learning rates over the 2 days [*F*(4, 89) = 0.80, *p* = 0.53]. The SZ group scored at “chance” in the first run and began to acquire the lexicon in the second run. This behavioral difference set the stage for the remaining acquisition of the lexicon and explained the main group effects [*F*(1, 22) = 7.82, *p* = 0.011]. Aside from this initial difference, the curves tracked each other for the duration of the experiment and performance plateaued for both groups at the same time. Reaction time data mirrored the accuracy data with both groups showing an expected decrease in reaction time for correct responses across 10 runs [*F*(2.8, 61.69) = 11.79, *p* = 0.0001], main effects of group with the HC group being faster overall [*F*(1, 22) = 18.43, *p* = 0.0001], and no interaction of run by group.

### Imaging Results

#### Behavior-PLS Analyses

The initial behavior-PLS analysis was conducted on both groups simultaneously. Three significant LVs emerged that characterized patterns of overlap and differences between brain regions, supporting accurate learning for both groups. Similar to a multivariate factorial ANOVA, these patterns represented group by learning run interactions and showed no main effects of group or learning run. To disentangle these interactions, we performed the analysis within each group, similar to how one would proceed to decompose an interaction effect in ANOVA.

**Figure 3:**
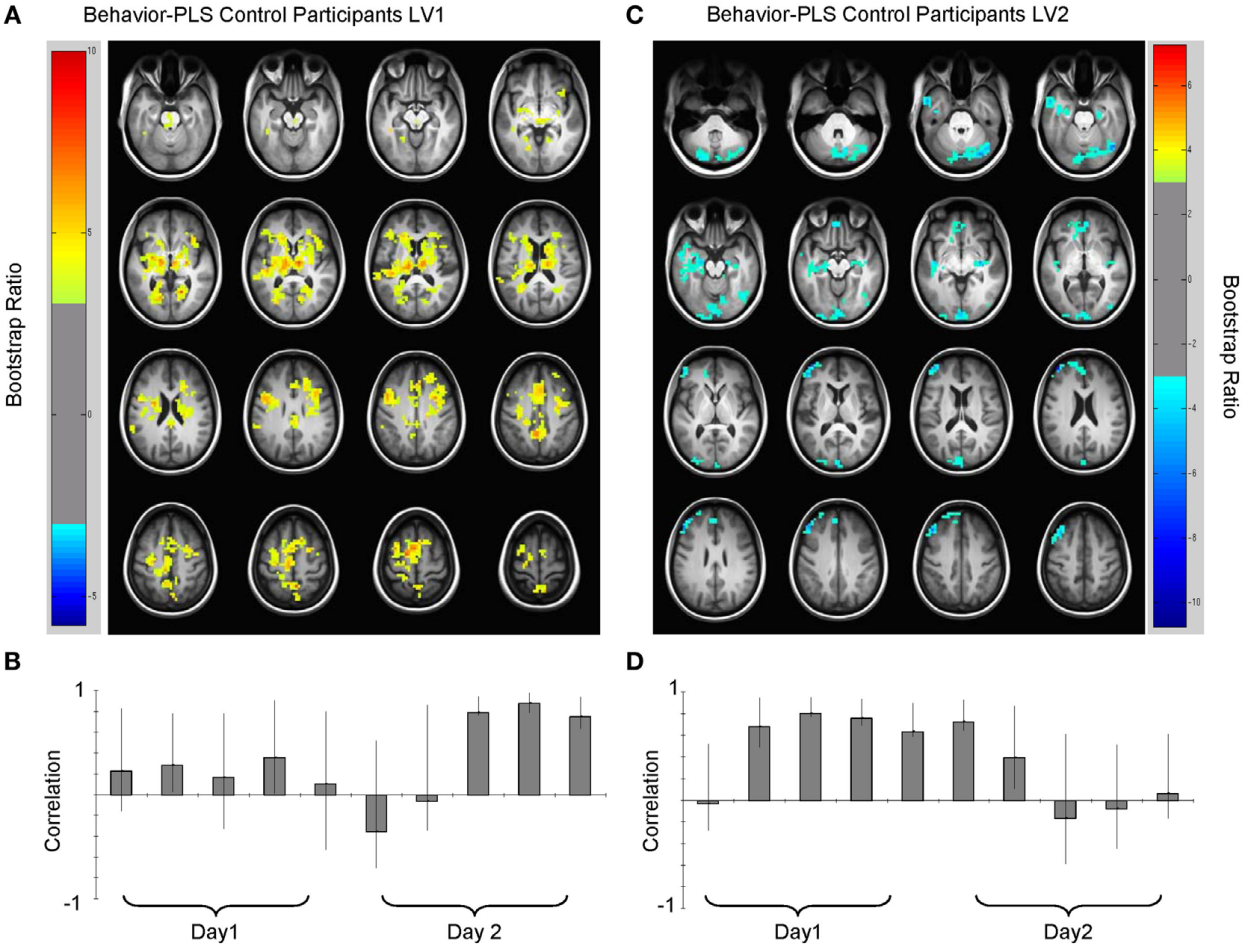
**Multivariate relationships between learning performance and blood oxygenation level-dependent (BOLD) signal for healthy control (HC) participants**. **(A)** Spatial pattern for late brain–behavior relationship (LV1, lag 2, see Table 2) showing brain regions engaged in late learning processes (learning runs 8 through 10) and **(B)** correlation magnitudes (Pearson *r*) for this pattern. HC with more BOLD activity relative to other HC in these regions were more successful learners. **(C)** Spatial pattern for early brain–behavior relationships (LV2, lag 5, see Table 3) showing brain regions engaged in early learning and the corresponding **(D)** correlation magnitudes (Pearson *r*) for each learning run in this multivariate pattern. Early in the experiment (runs 2 through 7), HC who had less relative BOLD activity in blue regions [negative bootstrap ratio (BSR)] had higher accuracy scores. Both early and late patterns had a permuted *p* < 0.0001. Error bars on the correlation bars are the bootstrapped 95% confidence intervals. Robust voxels are displayed at a BSR > ±3.

Analysis of the HC group data extracted two significant LVs that characterized the brain–accuracy relationship across all 10 learning runs (Figure 3; Tables 2 and 3). There was a clear demarcation for the HC group between the early (runs 2 through 7) and late (runs 8 through 10) brain patterns supporting accurate learning. The first LV emerged as the most statistically robust spatiotemporal pattern of all patterns for both groups [*p* < 0.000, SV = 188.9, crossblock covariation (CCV) = 24.6%] (Figure 3; Table 2). It characterized the brain–accuracy relationship for the last three learning runs. There was widespread brain distribution, with dominant areas including subcortical regions, particularly dorsal striatum and thalamus bilaterally, premotor regions, bilateral inferior frontal (opercularis and triangularis) regions, left fusiform gyrus, and cingulate cortices (anterior, mid, and posterior). Results should be interpreted as showing the relationship between engagement of brain areas covarying with performance. Therefore, control participants showing more relative activation in these regions at the end of the task were better learners overall.

**Table 2:** **Cluster peaks, coordinates, and bootstrap ratios (BSR) for first brain–accuracy network (LV1) for healthy control participants**.

Lag	MNI *X* (mm)	MNI *Y* (mm)	MNI *Z* (mm)	BSR	Cluster size (voxels)	Region
1	−5	−28	47	8.1	137	Left middle cingulate cortex
1	33	8	24	7.5	130	Right inferior frontal gyrus (pars opercularis)
1	−24	−18	51	6.2	87	Left supplementary motor area (SMA)
1	−58	−39	5	6.1	109	Left middle temporal gyrus
1	−40	15	27	5.8	85	Left inferior frontal gyrus (pars triangularis)
1	54	−21	7	5	56	Right superior temporal gyrus
2	−5	−12	57	8.2	801	Left SMA
2	−14	−24	−1	6.6	125	Left thalamus
2	33	−3	31	5.5	96	Right precentral gyrus
3	−41	−49	−24	10.3	1,293	Left fusiform gyrus
3	−6	6	63	8.8	2,250	Left SMA
3	40	−67	29	8.6	54	Right middle occipital gyrus
3	−10	−54	−41	5.8	57	Left cerebellum (IX)
4	−13	−13	−12	8.9	1,514	Left posterior thalamus
4	21	−61	1	8.1	72	Right lingual gyrus
4	6	−49	52	7.9	195	Right precuneus
4	−14	−72	−2	7.8	114	Left lingual gyrus
5	−26	−9	−11	8.9	931	Left putamen
5	−6	12	38	8.4	916	Left middle cingulate cortex
5	−33	−62	−40	6.9	110	Left cerebellum (crus 2)
5	3	−84	−2	6.4	57	Left calcarine gyrus
5	−27	19	8	5	62	Left insula lobe
6	−31	−1	54	8.7	1,652	Left precentral gyrus
6	7	28	21	5.7	155	Right anterior cingulate cortex
6	21	−51	−29	5.7	56	Right cerebellum (VI)
7	−17	−10	13	8.6	665	Left caudate
7	−1	4	49	8	219	Left SMA
7	−3	28	24	6.3	80	Left anterior cingulate cortex

*Lag refers to the period, in seconds, after stimulus onset during which the peak occurred. *X, Y*, and *Z* are the voxel coordinates of the peak in each cluster in MNI space. BSR represents each peak voxel’s PLS parameter estimate divided by its SE and is a measure of robustness. Positive BSR regions are areas where more blood oxygenation level-dependent activity relates to better accuracy and vice versa. A BSR threshold of 3 (roughly 99% confidence interval) was used as a cutoff to identify cluster peaks for this table. Cluster size refers to the number of contiguous voxels included in the cluster*.

The second HC LV robustly characterized the brain–accuracy relationship across runs two through seven (*p* < 0.000, SV = 176.1, CCV = 21.4%) (Figure 3; Table 3). This LV highlighted distributed areas used for the first phase of learning. Here, dominant areas for the pattern were left middle and left superior frontal, right inferior occipital regions, bilateral cerebellar, and bilateral hippocampi. Those with less relative activity in these areas demonstrated better learning performance. Further analysis focused on smaller voxel-clusters showed that those HC with greater bilateral opercular activity were more successful learners of the lexicon. In this LV, the first learning run did not contribute to the dominant pattern. As previously noted, for half of the first run, there was no operative learning principle given that this was the first exposure to a given correct pairing so here it is evident that there is a brain difference between a “guessing strategy” and a “learning strategy” *per se*.

**Table 3:** **Cluster peaks, coordinates, and bootstrap ratios (BSR) for second brain–accuracy network (LV2) for healthy control participants**.

Lag	MNI *X* (mm)	MNI *Y* (mm)	MNI *Z* (mm)	BSR	Cluster size (voxels)	Region
2	28	−93	−8	−8.6	298	R Inferior occipital gyrus
2	−38	12	49	−8.4	84	Left middle frontal gyrus
2	−24	−77	−52	−6.2	97	L cerebellum (IX)
3	−44	51	−12	−7.7	121	Left IFG (orbitalis)
4	28	−93	−8	−6	105	Right inferior occipital gyrus
5	−39	54	9	−9.7	147	Left middle frontal gyrus
5	52	−67	−24	−7.8	438	Right cerebellum (crus 1)
5	−52	2	−43	−7	175	L inferior temporal gyrus
5	32	−17	−13	−6.7	55	R hippocampus (Prob 70% that is CA)
5	−14	−85	−50	−6.6	110	L cerebellum
5	−23	62	10	−5.4	51	L superior frontal gyrus
5	−1	41	−28	−5.1	56	L medial orbital gyrus
7	14	−85	23	−7.4	148	R cuneus
7	8	−96	−11	−5.2	64	R lingual gyrus
7	−29	−78	−57	−4.7	52	L cerebellum

**Figure 4:**
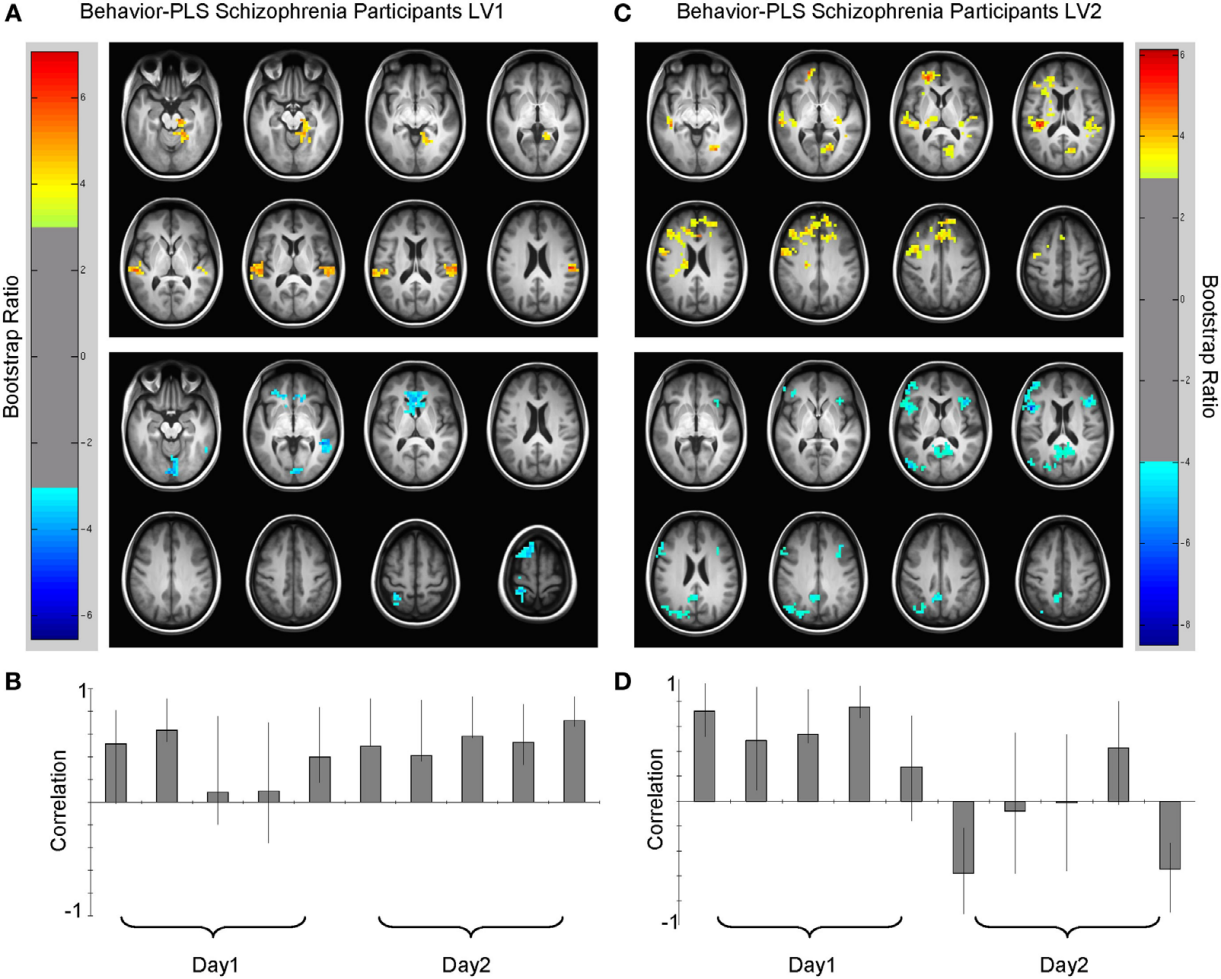
**Multivariate relationships between learning performance and blood oxygenation level-dependent (BOLD) signal for schizophrenia (SZ) participants**. **(A)** Spatial pattern for dominant brain–accuracy relationships that spanned both days of experiment with little modulation between days [LV1, *p* < 0.0001, crossblock covariation (CCV) = 23.3%] and **(B)** correlation magnitudes (Pearson *r*) for each learning run in this multivariate pattern. Warm colors (top panel, lag 3, see Table 4) illustrate brain regions engaged during learning where higher-performing participants with SZ have more BOLD activity. Cool colors (bottom panel, lag 7, see Table 4) show brain regions where poorer learners had more BOLD activity or conversely better learners had less relative BOLD activity. **(C)** Spatial pattern for secondary brain–accuracy relationships (LV2, *p* < 0.05, CCV = 16.3%) and **(D)** correlation magnitudes for this pattern (top panel, lag 1, Table 5 and bottom panel, lag 7, Table 5). Robust voxels are displayed at a bootstrap ratio > ±3.

Analysis of the SZ group data also extracted two significant LVs that characterized brain–accuracy relationships across all 10 learning runs (Figure 4). There was some separation between early and later learning, but the demarcation between these phases was less distinct than in the HC group without a clear shift to a pattern incorporating widespread dorsostriatal activation. Each LV captured some information about practice-related learning from start to finish. In contrast to the HC group, the first run did not separate out from the remaining runs in either of the LVs.

The first LV (*p* < 0.000, SV = 152.2, CCV = 22.3%) (Figure 4; Table 4) showed that BOLD variation in: bilateral superior and middle temporal gyri, bilateral middle occipital gyri, bilateral calcarine gyri, bilateral superior frontal, right hippocampal, and left caudate regions related to performance for the SZ group. In this pattern, more relative activation in bilateral superior temporal gyri, bilateral occipital gyri, and right hippocampal and parahippocampal areas related to greater learning accuracy. Those with less activity in caudate cortices compared to other participants with SZ were better learners of the lexicon. Additionally, there were differences in the directions of the correlations for some regions such as calcarine gyri, middle temporal gyri, and superior frontal cortices. More activity in left calcarine gyri and left middle temporal gyri and less activity in the same regions on the right correlated with better performance. In the superior frontal regions, more right-sided engagement and less left-sided engagement correlated with better learning performance. Similar to HC, secondary analyses that focused on smaller cluster sizes showed that in this LV bilateral opercular activity positively covaried with learning performance.

**Table 4:** **Cluster peaks, coordinates, and bootstrap ratios (BSR) for first brain–accuracy network (LV1) for participants with schizophrenia**.

Lag	MNI *X* (mm)	MNI *Y* (mm)	MNI *Z* (mm)	BSR	Cluster size (voxels)	Region
2	−32	−81	11	6.7	55	Left middle occipital gyrus
3	54	−19	20	4.8	61	Right superior temporal gyrus
3	−53	−30	4	4.1	52	Left superior temporal gyrus
3	16	−26	−23	4.1	63	Right parahippocampal gyrus
4	34	−75	24	4.9	129	Right middle occipital gyrus
4	−32	−82	6	3.8	51	Left middle occipital gyrus
5	−23	−71	7	5.7	93	Left calcarine gyrus
6	−9	47	8	−5.1	81	Left anterior cingulate gyrus
6	25	36	16	4.6	62	Right superior frontal gyrus
6	3	−29	19	3.9	62	Right posterior cingulate
7	55	−40	−9	−5.4	54	Right middle temporal gyrus
7	−5	5	2	−4.9	117	Left caudate (head)
7	−15	−40	72	−4.6	87	Left postcentral gyrus
7	11	−93	−3	−4.4	100	Right calcarine gyrus (area 17)
7	−14	18	55	−3.6	51	Left superior frontal gyrus

The second LV showed the brain–accuracy relationship primarily for the first four learning runs (*p* = 0.05, SV = 130. 00, CCV = 16.3%) (Figure 4; Table 5) and a pattern that differentiated between start and end of the study. There was an inverse relationship between the brain–behavior for this strategy at the start of the experiment vs. the end. Dominant areas for this pattern included primary auditory and visual cortices, anterior cingulate cortices, cerebellum, parahippocampal and hippocampal, bilateral opercular, and left middle frontal regions. In this pattern, participants with SZ who had more activity in primary auditory and visual regions and anterior cingulate cortices during the first four learning runs were better early learners. Conversely in this LV, less relative activation during the first four learning blocks in cerebellar, hippocampal, bilateral opercular, and left middle frontal regions related to better learning.

**Table 5:** **Cluster peaks, coordinates, and bootstrap ratios (BSR) for second brain–accuracy network (LV2) for participants with schizophrenia**.

Lag	MNI *X* (mm)	MNI *Y* (mm)	MNI *Z* (mm)	BSR (mm)	Cluster size (voxels)	Region
1	−35	−32	12	6.1	178	Left Heschl’s gyrus (TE 1.1)
1	−19	39	−4	5.8	328	Left anterior cingulate gyrus
1	34	−70	−7	5	86	hOC4v (extrastriate visual cortex)
1	−39	8	24	4.9	94	Left inferior frontal gyrus
1	50	−20	7	4.4	78	Right superior temporal gyrus (TE 1.0)
2	3	−52	−32	−4.9	412	Cerebellar vermis (9)
3	−49	10	3	−7.2	133	Left inferior frontal gyrus (pars opercularis)
3	47	22	1	−5.1	72	Right inferior frontal gyrus (pars triangularis)
3	−45	−82	14	−5	111	Left middle occipital gyrus
3	−2	−47	37	−4.2	110	Left precuneus
4	15	−55	15	−3.9	59	Right precuneus
5	60	14	24	−8.5	379	Right inferior frontal gyrus (pars opercularis and triangularis)
5	−10	−57	−60	−7.8	2,156	Left cerebellum (IX)
5	−62	−33	38	−6.6	315	Left supramarginal gyrus
5	−39	38	30	−5.5	401	Left middle frontal gyrus
5	−18	−60	−18	−6.2	86	Left cerebellum (VI)
5	33	−17	−23	−5.8	100	Right parahippocampal gyrus
5	−22	−26	−9	−5.6	85	L hippocampus
6	−14	−76	23	−6	99	Left superior occipital gyrus
6	−53	−47	43	−4.5	59	Left inferior parietal lobule
6	15	−56	10	−4.5	126	Right calcarine gyrus
6	16	−40	58	−3.8	57	Right postcentral gyrus (area 3b)
7	−14	−56	−56	−5.8	549	L cerebellum (IX)
7	−34	55	14	−5.4	57	L middle frontal gyrus
7	43	17	27	−4.3	56	R Inferior frontal gyrus (pars triangularis)

#### Brain–Behavior Patterns and the Relationship to Symptoms/Neuropsychological Tests

**Figure 5:**
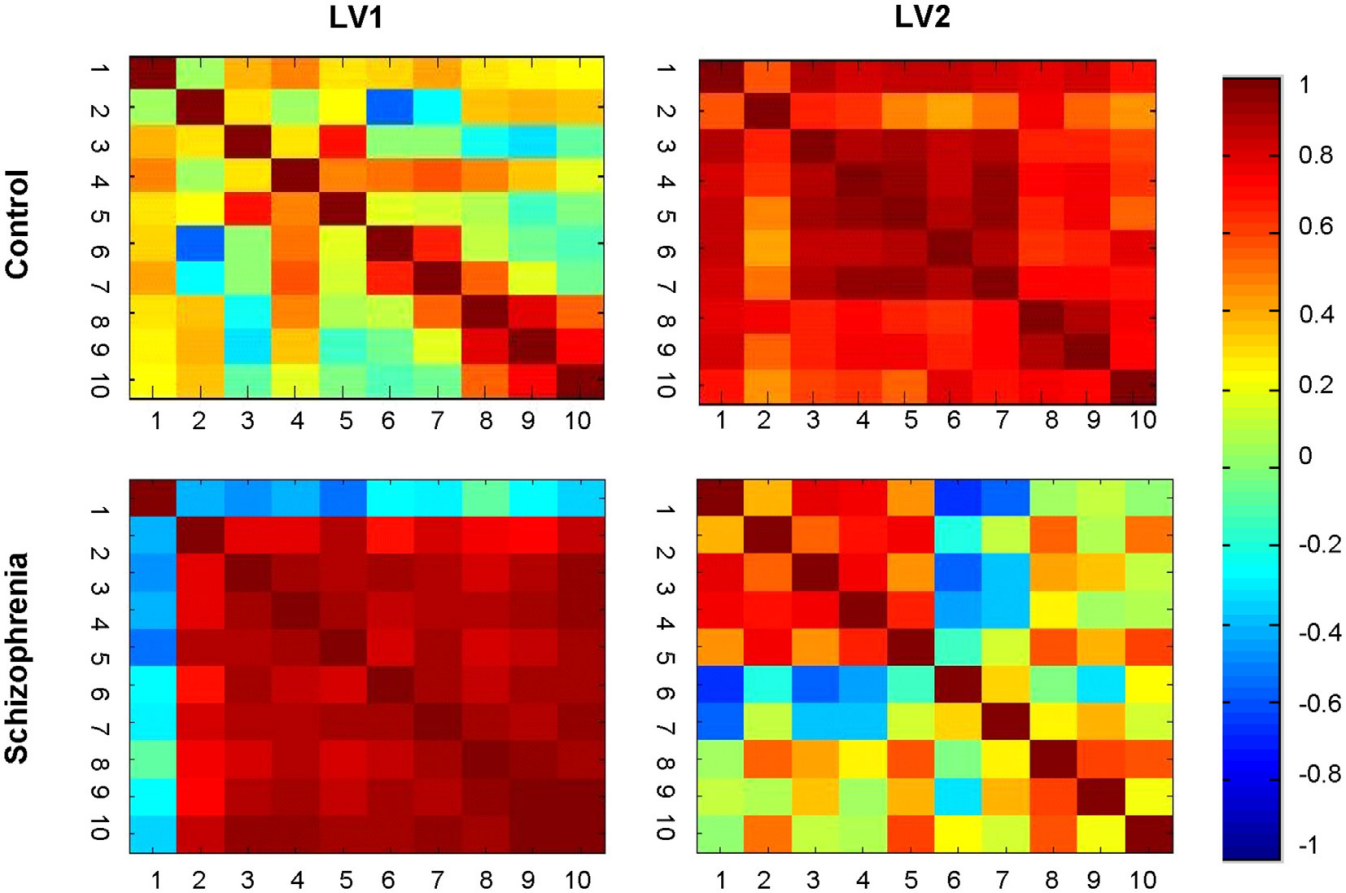
**Brainscore correlation matrices**. Correlation of brainscores from the brain behavior-PLS analysis within each group. Healthy controls showed two distinct patterns: covariation clustering in the late learning phases (LV1) and general correlations across learning (LV2). The schizophrenia group showed a similar general pattern (LV1), but the secondary pattern mapped too early, rather than late, learning (LV2).

The brainscore matrices (Figure 5) illustrate the consistency of spatial patterns within each group. The HC show two distinct patterns characterizing movement through the experiment. The brainscore matrix for the second LV (top right) shows a high correlation between brainscores across all 10 learning runs. In the first LV (top left), in contrast, there is a high intercorrelation for brainscores from runs 8 through 10 that does not correlate with preceding runs, which could indicate a shift in learning toward another underlying process (e.g., automatization). The pattern for LV1 (bottom left) for the SZ group is similar to the correlation matrix for the LV2 for the controls, with high correlation between brainscores across all runs. Again, this suggests consistent movement through the two experimental days for individuals in the SZ group manifesting this brain–behavior pattern. The bottom right matrix (LV2) for the SZ group illustrates an alternative brain–behavior relationship manifest during the first learning day that did not substantively carry over into the second day of learning.

Table 6 lists the correlation between brainscores and neuropsychological and symptom scores. There were significant relationships between the brainscores in LV1 for the SZ group in total RBANS scores and all subscales of the RBANS (excluding attention) at the 95th percentile. In other words, those with SZ who performed better on cognitive measures, particularly language, were more likely to demonstrate this brain–behavior relationship when learning accurately. Symptom scores were significantly correlated (weakly) with brainscores, but only when taken in total. Thus, those with a higher level of symptomatology tended also to activate this network of regions to learn accurately. The brainscores for LV2 for the SZ group showed no significant correlations with any of the neurocognitive or symptom measures. Notably, there was also no significant correlation between brainscores for either LV or antipsychotic medication dosage, suggesting that the brain–behavior relationships were not explainable by medication effect.

**Table 6:** **Correlation between brainscores for first LV for each group and neurocognitive and clinical measures**.

Measures	Control group brainscores (LV1)		Schizophrenia group brainscores (LV1)	
	*r*	95% CI	*r*	95% CI
Attention	0.11	(−0.18 to 0.52)	0.22	(−0.53 to 0.58)
Immediate memory	**0.63**	**(0.27 to 0.87)**	**0.41**	**(0.19 to 0.74)**
Delayed memory	0.06	(−0.42 to 0.59)	**0.34**	**(0.02 to 0.60)**
Language	0.23	(−0.07 to 0.53)	**0.51**	**(0.07 to 0.78)**
Visuospatial construction	−0.04	(−0.53 to 0.48)	**0.49**	**(0.15 to 0.81)**
Battery for the Assessment of Neuropsychological Status total	**0.45**	**(0.15 to 0.72)**	**0.48**	**(0.18 to 0.78)**
Positive and Negative Syndrome Scale (PANSS) general	n/a	n/a	0.37	(−0.23 to 0.66)
PANSS negative	n/a	n/a	0.01	(−0.76 to 0.41)
PANSS positive	n/a	n/a	0.34	(−0.36 to 0.62)
PANSS total	n/a	n/a	**0.36**	**(0.02 to 0.81)**
Medication (cpz equivalent)	n/a	n/a	0.16	(−0.8 to 0.84)

*Values presented in bolded font are statistically significant at the 95% confidence interval*.

The HC group showed a very different relationship between brainscores and neurocognitive measures. In the LV1, there was a significant relationship of moderate strength between brainscores and immediate memory [*r* = 0.63, CI_95_ = (0.27–0.87)] and between brainscores and total RBANS [*r* = 0.45, CI_95_ = (0.15–0.72)] at the 95th percentile. In other words, HC who scored higher in general on cognitive measures, and particularly on measures of immediate (working) memory, were more likely to demonstrate this brain–behavior relationship. None of the other individual cognitive domains were significantly correlated. Lastly, there were no significant correlations between the brainscores of LV2 and any of the RBANS scores.

### Summary of Results

Both participant-groups benefited from practice in that they learned the lexicon. The differences between the behavioral learning curves of the two groups can primarily be explained by the first two runs. While the HC showed accuracy levels beyond chance in this first run, the SZ group did not. Beyond this point, the two groups demonstrated learning at the same rate over the course of the 2 days.

The most salient finding was that although there was anatomical overlap between the brain areas engaged for successful lexicon learning for both groups, the timing of this engagement and relative importance of these regions differed markedly between the groups. The HC group showed a clear and robust differentiation in brain patterns for successful lexicon learning between early and late stages of the experiment that were not manifest in the SZ group despite comparable behavioral performance and a similar plateauing of performance at the end of the experiment.

For the HC group, practice of the learning task over the 2 days lead to a clear transition toward a unique network of brain activity supporting successful learning for the last three runs of the experiment. The HC pattern at this late stage was widely distributed and showed regional overlap with the pattern supporting learning earlier in the experiment for the HC. However, early- vs. late-learning differences were dominated by widespread increased engagement in subcortical areas, particularly dorsostriatal and thalamic regions. At this later stage, more relative engagement of these areas related strongly to more accurate learning. Furthermore, the brainscores at this learning stage did not correlate with those at an earlier stage, suggesting that this pattern represented a fundamental shift in the brain–accuracy network. Lastly, the HC who were most likely to manifest this late pattern were those who scored highest on cognitive estimates of working memory abilities out of the scanner.

In contrast, the SZ group showed a different brain–accuracy relationship that did not transition from “early learning” to a clearly demarcated “late learning” phase. Instead, the SZ group showed patterns that differentiated successful lexicon learners on the basis of relative engagement of secondary sensory cortices involved in perceptual processing and persistent medial–temporal engagement. While bilateral regions were engaged in the brain–accuracy networks in SZ, better learners showed greater laterality in a number of regions. Additionally, persons in the SZ group were more likely to manifest this relationship if they scored higher on cognitive measures and displayed more symptoms of SZ (*via* the total PANSS score). This second finding may seem non-intuitive; however, it suggests that this particular brain–behavior relationship represents a coherent compensatory strategy specific to SZ *per se*.

## Discussion

The multisession design of our study as well as the multivariate brain–behavior analysis allowed us to observe the systems supporting practice-related lexicon-learning *in vivo*. The two dominant findings include differences between early- and late-learning brain engagement patterns related to successful learning for each group. Compared to the HC group, relative engagement in perceptual processing and persistent medial–temporal lobe engagement played a larger role in driving successful learning for the participants with SZ.

The HC participants engaged globally distributed cortical and subcortical regions to support accurate lexicon learning throughout the experiment. Prefrontal regions, including left-lateralized middle, superior frontal, and medial orbitofrontal regions, were engaged in early learning processes, but were but were not part of the brain–accuracy relationship in the late stages for the controls. After a period of practice, behavior metrics plateaued and the HC shifted into a clearly demarcated stage of late learning at the end of the second day of scanning. The brainscore matrices in Figure 5 support this transition suggesting a fundamental shift in underlying processes when engaging this late brain–accuracy network.

In this late learning phase, neostriatal (caudate and putamen) engagement differentiated better from worse learners in the HC. The neostriatum is crucial for the incremental learning of associations through practice that underlie both motor and non-motor habit learning (10). A recent line of inquiry suggests that the associative (caudate and anterior putamen) and the sensorimotor (putamen) striatum have different roles in learning and the shift from effortful learning toward automaticity. The associative striatum is active in early learning and then activity gradually diminishes; the sensorimotor striatum becomes engaged in the transition from goal-directed to automatic or habit-like performance [see Ref. (12) for review]. Taken from this perspective, the latent variable characterizing late learning in our experiment seems to be capturing the transition from goal-directed to automatic performance. The experimental design does not allow an absolute conclusion that automaticity has occurred, but given the brain–behavior results this interpretation is reasonable.

There are further reasons to suggest that this LV captures brain–behavior relationships as the HC *shift* toward late-learning processes in our experiment. Here, as noted, key domain-general prefrontal regions fall away in importance, while other components of the domain-general control network in practice-related learning (e.g., anterior and middle cingulate cortices, bilateral lingual gyri) persist (14). Medial–temporal regions also followed a pattern of engagement consistent with early- vs. late-learning processes in the relational-learning literature (44). Lastly, the emergent contralateral SMA-accuracy engagement at this stage is consistent with studies that have shown sharp increases in SMA just as participants’ transition to automaticity in motor-learning paradigms (45).

The thalamus emerged late in the brain–accuracy network in the HC group. As a relay station with both reciprocal and unidirectional connections between cortical and subcortical structures, the thalamus plays an important role in sensory processing, motor and cognitive learning paradigms, including language and lexicon learning (46–48). The thalamus consistently emerges in later learning stages in the non-declarative motor-learning literature (49). A study looking at early vs. late changes in simple motor-learning task with a brief period of practice echoed many of our findings with the striatum, cingulate, and thalamic activity providing the most dissociation between early and late learning (50). The role of the thalamus in the language and lexicon-learning literature is still under debate. However, in general, cortico–thalamic interactions are thought to mediate phonologically based knowledge driven lexical processes whereas thalamic–basal ganglia interactions drive general rule-based learning, less specific to language *per se* (47, 51). Taken in total, the emergence of thalamus, putamen, and SMA and the reduction in prefrontal engagement in this late learning brain–accuracy network suggests a shift away from explicit effortful learning processes toward procedurally driven automatic processes for the HC, but not the SZ group.

Despite the same amount of inter-experiment practice, similar learning rates and a plateauing of performance at the end of the experiment, the SZ group did not transition to the same demarcated late-learning brain–accuracy network demonstrated by the HC group. These differences in cortico-subcortico-thalamic-cerebellar circuit engagement between the two groups can be contextualized within the “cognitive dysmetria” model of SZ (52, 53). As per this model, disruptions in distributed cortico-thalamic-cerebellar circuitry account for many of the observed cognitive deficits in the disorder, including those relying on planning, precise timing, updating, and coordination of input for learning purposes. Our results are in line with these findings. Consistent with the model, the SZ group did not transition to a brain–accuracy network that engaged key subcortical, thalamic, and cerebellar regions as in the high-performing controls with the best working memory capacities. Evidence is accumulating for the thalamus as a key node in this brain–learning network in SZ (54). Studies have demonstrated thalamic hypoconnectivity between frontal and striatal regions and hyperconnectivity in sensory, somatosensory, and motor regions in chronic, early, and at-risk for SZ groups (55–58). Additionally, recent anatomical studies suggest a relationship between (decreased) thalamic volume and language learning skills in SZ who has been heretofore underappreciated (59). Our results suggest that aberrant functional thalamic engagement also impacts practice-related lexicon-learning processes in SZ, but further connectivity analyses are needed to fully characterize the relationship.

Instead of the early- vs. late-learning patterns observed in the HC, the SZ participants show a dominant brain–behavior relationship supporting accurate learning that spanned all 10 learning runs. Rather than a transition toward subcortical-thalamic-cerebellar engagement, better learners with SZ showed persistent engagement of medial–temporal lobe structures, more engagement of bilateral auditory and visual processing cortices and persistent superior frontal engagement in the brain–accuracy network. The pattern suggests that for the SZ group at this stage there was brain engagement consistent with effortful learning while moving through the entire experiment, and there was no transition from early frontal–hippocampal mediated processes to later subcortically mediated ones even as behavior plateaued. The correlation between this brain–accuracy network and better performance on a number of cognitive measures suggests that this represents a compensatory strategy specific to the SZ group.

Our lexicon-learning task mimics how one might learn a second language in real-life. Initial learning was explicit with instructions given to subjects that they would be trying to learn a new vocabulary. Encoding would therefore rely on declarative mechanisms as participants effortfully attended to the stimuli. As the task progressed, there was no feedback given, rather there was reliance on statistical co-occurrence with correct-pairings occurring more frequently than incorrect pairings. Initially, the HC group engaged medial–temporal and prefrontal regions to learn the lexicon in the early-learning stages, with better learners needing to engage these regions less (Figure 3). Here, less engagement covarying with better performance is likely due to early learning, reduced novelty, and efficient encoding as HC quickly begin to shift toward non-declarative processes. This parallels the findings of Breitenstein et al. who originally developed this lexicon-learning task and measured BOLD signal changes across 1 day of training in HC (35).

The situation with the SZ group was quite different in these regions. The hippocampus was relevant in the first (most robust) brain pattern that spanned all 10 of the learning runs. Unlike in the HC, the hippocampus did not disengage at the end of the early stage. In this pattern, persistent engagement related to better learning performance. More parahippocampal engagement in this latent variable also related to better cognitive performance out of the scanner suggesting that here these medial temporal regions were persistently engaged in higher-performing participants with SZ to compensate for inefficient early learning, more effortful or prolonged processing (60). Certainly, deficits in declarative memory are well documented in SZ, with particular impairments in verbal memory (61, 62). There seems to be particular challenges in relational (vs. item) learning and these impairments have been shown to relate to impairments in both dorsolateral prefrontal cortical and hippocampal activation (19). Consistent with the meta-analytic findings of Ragland et al., the increased engagement of medial temporal regions in this key brain–accuracy network for the SZ participants in our study may also reflect compensation for the notable absence of dorsolateral and medial orbital prefrontal regions in this brain–accuracy network (63). While this compensatory process allowed some SZ participants to learn the lexicon more accurately, it did not lead to a transition to the striatal-thalamic-cerebellar engagement as seen in the HC participants.

The second latent variable for the SZ group captured an alternative early-learning process with spatial overlap in some regions with the early successful learning networks of the HC group. Similar to the HC group, in this network less relative activity in the HPC related to better learning performance. However, in the SZ group the pattern did not carry over into the second day as it did in the HC group suggesting that for the SZ group, this brain–behavior pattern does not lead to “buildable” learned associations (see correlation matrix figure). The two brain–accuracy relationships highlight the importance of neural context (64) by showing that medial temporal regions subserve different roles depending on the network in which they are embedded. While both of the patterns support more accurate learning in SZ, only the first does so in a tractable way that carries through the experiment.

Bilateral auditory and visual sensory processing areas were key regions in the dominant brain–accuracy pattern for the SZ group; those who had more relative activity in auditory and visual perceptual processing regions were more accurate learners. Another way to consider this finding is that, for the SZ group, there were degrees of freedom in perceptual processing areas for the SZ group and those who could leverage activity in these areas could compensate and performed better whereas those who could not engage these regions were worse learners. These same regions played a similar, but much less dominant, role for the controls in late learning only. Thus, given the nature of the task, it is likely that all the controls were functioning optimally in processing and integrating incoming stimuli early in the task whereas the SZ group showed more variability in this capacity.

Auditory processing dysfunction is a common (but often underappreciated) impairment in SZ with broad downstream consequences for symptomatology, psychosocial and cognitive functioning [see Ref. (65) for review]. Strongest evidence comes from neurophysiological studies showing tone matching abilities *via* reduced mismatch negativity (MMN) event-related potentials (ERPs). These ERPs are elicited when an auditory stimulus is different from what is anticipated given prior stimuli (e.g., auditory “oddball” paradigms). Reduced MMN is seen in SZ, in those at-risk for developing psychosis and when psychosis is induced by ketamine (a NMDA-receptor antagonist) (66, 67). Studies also show impaired auditory gating at a basic filtering level (*via* PPI and P50 gating) in persons with SZ and their family members (68, 69). Less attention has been paid to visual processing in SZ, but evidence suggests that impairments in processing visual stimuli and integrating them with auditory cues are a frequent finding in the disorder (68, 70). In our study, the event markers were set to picture offset which means not measuring picture detection, but rather the processing and relational integration of the incoming perceptual information. The SZ group who showed more engagement in bilateral MOG (vs. unilateral in the HC) were more successful learners. Thus, those who were able to increase relative activity in bilateral perceptual processing regions in both auditory and visual regions relative to other participants with SZ while practicing the task were better lexicon-learners overall.

Given the potential impact of antipsychotic medications on brain findings in cognition (71, 72), it is important to highlight the lack of antipsychotic medication effect that we found. Given our cohort, though, we are unable to fully disentangle the role that antipsychotic medications play in the brain–accuracy networks; however, we were able to establish that the amount of medication did not drive our findings. Other limitations include the small size of our final sample. Given this, results should be viewed as preliminary and validated through replications with a larger sample in the future. That said, a number of mitigating factors including the robustness of our findings, the use of resampling statistics and direct brain–behavior measurements make the results, as they stand, compelling. Lastly, there were behavioral differences between groups on the learning task thus some of the results may be specific to performance rather than solely disease state. However, our analysis also examined the variations supporting accuracy for each group independently thus relying on within-group variance lessens the impact of performance differences.

In conclusion, our results point to differences in the network transitions from early to later learning in SZ. Where the HC participants moved quickly away from a brain engagement pattern suggestive of effortful explicit learning processes and toward subcortically driven implicit ones, the SZ participants did not. This difference was in spite of similar overall learning rates. These results give insight into how, in more challenging learning situations, the capacity to successfully integrate new information and skills could potentially fail in SZ. On the other hand, going forward our findings may also help construct estimates of learning potential for various rehabilitation strategies. For instance, bottom-up neuroplasticity-based cognitive rehabilitation such as auditory training in which there is repeated training in processes such as basic tone discrimination, has been shown to be a very promising remediation treatment for cognitive deficits in SZ (73). However, there is significant heterogeneity in individual response to this treatment (74, 75). Our results both shed light on the possible underlying brain–behavior mechanisms in responders and may also, in the future, serve as a predictive biomarker for rehabilitation potential. Those who manifest the brain–behavior relationship characteristic of better learners in our experiment, with more relative engagement in auditory processing regions may also be more likely to respond to auditory-targeted cognitive training and other neuroplasticity-based remediation programs.

## Ethics Statement

The study was approved by two ethics committees: (1) The Research Ethics Board (REB) of the CAMH and (2) The REB of Baycrest Health Sciences. Both are teaching hospitals affiliated with the University of Toronto. All participants provided written informed consent. Some of the participants in this study had a diagnosis of SZ. Care was taken at each study visit to assess for any changes in mental status that would preclude safe participation in the study. This assessment was done by a psychiatrist specializing in SZ.

## Author Contributions

Author contributions to the article were as follows: (1) conception and design of the work: MK, GR, and AM; (2) data collection: MK; (3) data analysis and interpretation: MK and AM; (4) drafting the article: MK and AM; (5) critical revision of the article: MK, GR, and AM; (6) final approval of the version to be published: MK, GR, and AM; (7) agreement to be accountable for all aspects of the work including appropriate investigation and resolution of all questions related to the accuracy and/or integrity of any part of the work: MK, GR, and AM.

## Conflict of Interest Statement

The authors declare that the research was conducted in the absence of any commercial or financial relationships that could be construed as a potential conflict of interest.

## Footnotes

^1^http://www.curdes.com.

^2^http://www.pstnet.com.

^3^http://afni.nimh.nih.gov/afni.

^4^http://bishopw.loni.ucla.edu/AIR5/.

^5^www.fmrib.ox.ac.uk/fsl.

^6^http://www.fil.ion.ucl.ac.uk/spm/.
